# Correction: Effects of alcohols as sacrificial reagents on a copper-doped sodium dititanate nanosheets/graphene oxide photocatalyst in CO_2_ photoreduction

**DOI:** 10.1039/d4ra90098g

**Published:** 2024-09-11

**Authors:** Nutkamol Kitjanukit, Wannisa Neamsung, Apisit Karawek, Napat Lertthanaphol, Napatr Chongkol, Koki Hiramatsu, Tomoya Sekiguchi, Soraya Pornsuwan, Takahiro Sakurai, Woranart Jonglertjunya, Poomiwat Phadungbut, Yuichi Ichihashi, Sira Srinives

**Affiliations:** a Department of Chemical Engineering, Faculty of Engineering, Mahidol University Nakhon Pathom 73170 Thailand sira.sri@mahidol.ac.th; b Department of Chemical Science and Engineering, Graduate School of Engineering, Kobe University Rokkodai-cho 1-1, Nada Kobe 657-8501 Japan; c Department of Chemistry, Faculty of Science and Center of Excellence for Innovation in Chemistry, Mahidol University Bangkok 10400 Thailand; d Research Facility Center for Science and Technology, Kobe University Rokkodai-cho 1-1, Nada Kobe 657-8501 Japan

## Abstract

Correction for ‘Effects of alcohols as sacrificial reagents on a copper-doped sodium dititanate nanosheets/graphene oxide photocatalyst in CO_2_ photoreduction’ by Nutkamol Kitjanukit *et al.*, *RSC Adv.*, 2024, **14**, 27980–27989, https://doi.org/10.1039/D4RA04585H.

The author regrets that the funding information was incorrectly shown in the Acknowledgements section of the original manuscript. The corrected Acknowledgements are as shown below.

Sira thanks the MU Talent Program, Faculty of Engineering, Mahidol University, and the Malaysia-Thai Joint Authority (MTJA) (Research CESS fund) for financial support. Sira is grateful to staff from the Mahidol University Frontier Research Facility (MU-FRF) and the Department of Chemical Engineering Central lab for material characterizations and photoreduction product analysis. Sira credits Graham K. Rogers for his contribution to manuscript editing. Nutkamol acknowledges the 60th Year Supreme Reign of His Majesty King Bhumibol Adulyadej Scholarship and a scholarship from the postgraduate mobility program for financial support. Nutkamol conveys her gratitude to the Research Facility Center for Science and Technology staff at Kobe University for their kind assistance and hospitality during her stay.

The Royal Society of Chemistry apologises for these errors and any consequent inconvenience to authors and readers.

